# A Review of Major Patents on Potential Malaria Vaccine Targets

**DOI:** 10.3390/pathogens12020247

**Published:** 2023-02-03

**Authors:** Reysla Maria da Silveira Mariano, Ana Alice Maia Gonçalves, Diana Souza de Oliveira, Helen Silva Ribeiro, Diogo Fonseca Soares Pereira, Ingrid Soares Santos, Daniel Ferreira Lair, Augusto Ventura da Silva, Alexsandro Sobreira Galdino, Miguel Angel Chávez-Fumagalli, Denise da Silveira-Lemos, Walderez Ornelas Dutra, Rodolfo Cordeiro Giunchetti

**Affiliations:** 1 Laboratory of Biology of Cell Interactions, Department of Morphology, Institute of Biological Sciences, Federal University of Minas Gerais, Belo Horizonte CEP 31270-901, MG, Brazil; 2 Laboratory of Biotechnology of Microorganisms, Federal University of São João Del-Rei, Divinópolis CEP 35501-296, MG, Brazil; 3 Computational Biology and Chemistry Research Group, Vicerrectorado de Investigación, Universidad Católica de Santa María, Urb. San José S/N, Arequipa 04000, Peru; 4 Campus Jaraguá, University José of Rosário Vellano, UNIFENAS, Belo Horizonte CEP 31270-901, MG, Brazil

**Keywords:** malaria vaccine, patents, stage of development

## Abstract

Malaria is a parasitic infection that is a great public health concern and is responsible for high mortality rates worldwide. Different strategies have been employed to improve disease control, demonstrating the ineffectiveness of controlling vectors, and parasite resistance to antimalarial drugs requires the development of an effective preventive vaccine. There are countless challenges to the development of such a vaccine directly related to the parasite’s complex life cycle. After more than four decades of basic research and clinical trials, the World Health Organization (WHO) has recommended the pre-erythrocytic Plasmodium falciparum (RTS, S) malaria vaccine for widespread use among children living in malaria-endemic areas. However, there is a consensus that major improvements are needed to develop a vaccine with a greater epidemiological impact in endemic areas. This review discusses novel strategies for malaria vaccine design taking the target stages within the parasite cycle into account. The design of the multi-component vaccine shows considerable potential, especially as it involves transmission-blocking vaccines (TBVs) that eliminate the parasite’s replication towards sporozoite stage parasites during a blood meal of female anopheline mosquitoes. Significant improvements have been made but additional efforts to achieve an efficient vaccine are required to improve control measures. Different strategies have been employed, thus demonstrating the ineffectiveness in controlling vectors, and parasite resistance to antimalarial drugs requires the development of a preventive vaccine. Despite having a vaccine in an advanced stage of development, such as the RTS, S malaria vaccine, the search for an effective vaccine against malaria is far from over. This review discusses novel strategies for malaria vaccine design taking into account the target stages within the parasite’s life cycle.

## 1. Introduction

Malaria is an infectious disease caused by *Plasmodium* spp. parasites, transmitted by bites from infected female *Anopheles* mosquitoes. According to the World Health Organization (WHO), globally, there were an estimated 247 million malaria cases in 2021 in 84 malaria endemic countries (including the territory of French Guiana), an increase from 245 million in 2020, with most of this increase coming from countries in the WHO African Region [1,2]. To control the disease more effectively, different strategies have been employed to control the disease, but the ineffectiveness in controlling vectors and parasite resistance to antimalarial drugs suggest that the development of a vaccine as a preventive measure could be important [3]. Currently, the main pillars for the management of malaria are rapid diagnostic tests and artemisinin derivatives for treatment. However, these strategies have not been enough to maintain a downward trend in malaria incidence and mortality. Recent findings in the pathophysiology of malaria have highlighted the importance of the host’s response to the infection [4].

*Plasmodium vivax*, *Plasmodium falciparum*, *Plasmodium malariae*, and *Plasmodium ovale* are among the species that commonly affect humans. *P. falciparum* is responsible for the most severe form of disease and death and is more prevalent in Africa. The second most common species is *P. vivax*, found in South and Southeast Asia, Central and South America, and some countries in Europe and North Africa [2]. The cycle begins with the pre-erythrocytic phase in which the mosquito performs the blood meal by inoculating sporozoites in human host’s bloodstream. Sporozoites actively reach the peripheral vascular system and migrate to the liver where they replicate in hepatocytes, forming merozoites that are released into the bloodstream. Merozoites invade red blood cells (RBCs), initiating the erythrocytic phase, and develop through the ring, trophozoite, and schizont stages before forming new merozoites that are released at the exit of the schizonts and reinfect new RBCs. A small number of the blood-stage parasites develop in sexual stages called gametocytes, which pass through microvasculature in the dermis where they are captured by another mosquito [5]. After fertilization and sporogonic development in the gut of the mosquito, infectious sporozoites are formed and reach the salivary glands to be transmitted to another host [5,6]. Intervention strategies, including vaccines, are predicted to be most effective if targeted to specific stages of the parasite’s life cycle and/or directed to proteins expressed in those stages [7]. Several technologies involving the development of malaria vaccines using different formulations with *Plasmodium* antigens or immunogenic fragments have been reported (US5112749A, US20160038580A1, EP1544211A1, US20190374629A US20040137512). Due to the complexity of the *Plasmodium* biological cycle, most vaccines reported against malaria have more than one target antigen. This review reports the patent landscape for malaria vaccines using different target stages.

## 2. Search Strategy and Selection Criteria

The following patent databases were consulted: European Patent Office (Espacenet—https://worldwide.espacenet.com (accessed on 4 May 2022)), United States Patent and Trademark Office (USPTO—https://www.uspto.gov/patents-application-process (accessed on 4 May 2022)), United States Latin America (LATIPAT—https://lp.espacenet.com/ (accessed on 4 May 2022)), and Patentscope -Search International and National Patent Collections (WIPO—https://patentscope.wipo.int (accessed on 4 May 2022)). The keywords used were: “malaria vaccine” AND “pre-erythrocytic” OR “sexual” OR “blood”. The titles and abstracts from pre-selected articles were read and evaluated by two independent researchers.

## 3. Results

**Figure 1:**
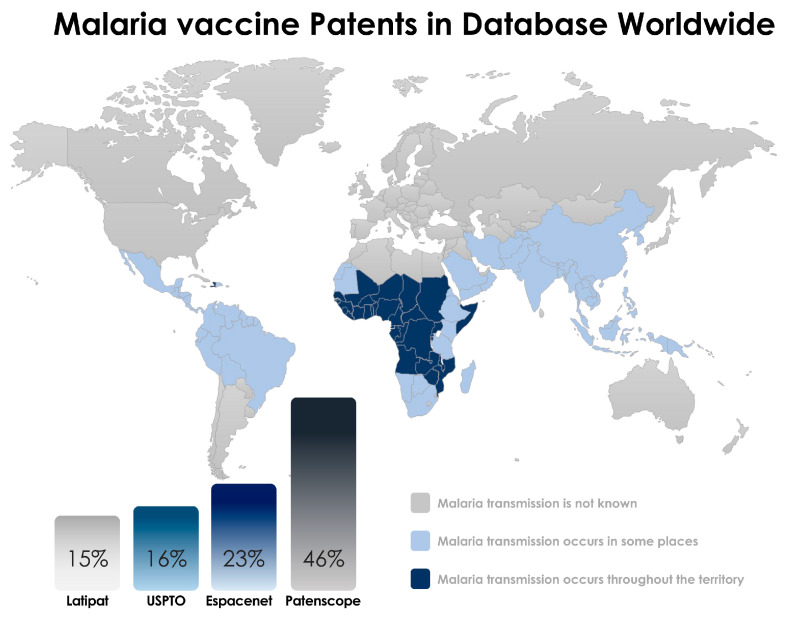
Current status of malaria vaccines found in the patent database. Data were collected through research at the European Patent Office (Espacenet), United States Patent and Trademark Office (USPTO), United States Latin America (LATIPAT), Patentscope -Search International, and National Patent Collections (WIPO).

**Figure 2:**
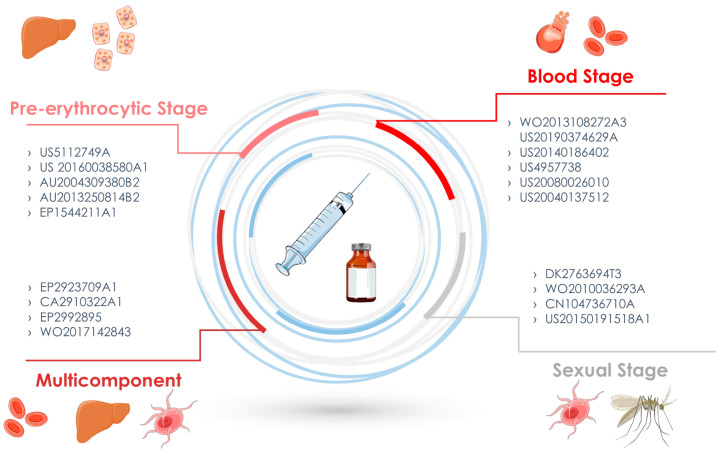
Malaria vaccine inventions according to the target phase, pre-erythrocytic phase, blood phase, sexual phase, or multiple stages.

In the end, a total of 44 patents in the Patentscope, 14 patents in the Espacenet, 15 patents in the USPTO, and 22 patents in LATIPAT were identified (Figure 1). The abstracts were read and the selected patents are described in this review. The selected inventions are described in Table 1 according to the three stages that define a possible candidate for a vaccine against malaria: the pre-erythrocytic stage, the blood stage, and the sexual stage, in which the target is the form of sexual or gametocyte parasites (Figure 2). This review takes a close look at the technologies for developing malaria vaccines according to published papers, especially those covering patents.

### 3.1. Challenge of Developing a Malaria Vaccine

Although the development of malaria vaccines began more than thirty years ago, there is no commercially available vaccine. Currently, with the appearance of COVID-19, there has been a significant decline in the use of long-lasting insecticide nets, indoor residual spraying, seasonal malaria chemoprophylaxis campaigns, access to rapid diagnostic tests, and effective malaria treatment, all of which can have a consequential impact on the mortality rate [8,9]. There are numerous challenges to be faced with developing a malaria vaccine that are directly related to the parasite’s immunobiology. The genome of *P. falciparum* is highly complex and is distributed on fourteen chromosomes that express more than 5000 separate genes [10]. The genetic diversity most likely contributes to the parasite’s ability to adapt to the immune systems of its human and mosquito hosts [11]. In addition, other difficulties have hindered the development of a vaccine, such as the substantial specificity of the phase of expression of the antigen by *Plasmodium* parasites, meaning vaccines that are candidates for one stage of the life cycle are unlikely to have an effect on another stage [12]. Finally, the target species *P. falciparum* and *P. vivax* are not able to infect small animals or Old World macaques, thus excluding the most widely used animal models for a direct evaluation of the vaccine [13].

Other malaria parasites infect these species, such as *Plasmodium berghei,* an important rodent malaria model [14,15], and *Plasmodium chabaudi*, *Plasmodium yoelii*, and *Plasmodium vinckei*, which naturally infect mice [16]. Although non-human primates are a valuable resource for testing vaccine candidates, they are not found naturally infected but can be easily infected with *P. falciparum* or *P. vivax*, such as *Aotus* genus, largely used in malaria research [17], and *Rhesus* macaques infected with *P. falciparum*, also used as models to assess cerebral malaria [18]. Although mice and non-human primates afford many advantages for the study of malaria, they differ substantially from human parasites [19]. Some limitations have to be considered, such as the lack of immunological tools to assess the immune response of non-human primates and the mistranslation of antigens that are protective in mouse models for human malaria, indicating a pressing need to improve experimental models [13].

The most advanced candidate to-date is the pre-erythrocytic *P. falciparum* (RTS, S) vaccine (trade name Mosquirix) [19]. The most notable results described for RTS, S vaccine were: (i) 34% efficacy with significant protection against natural *P. falciparum* infection [20], (ii) safety and immunogenic in infants [21], and (iii) a three-dose vaccination with RTS, S was protective against clinical malaria [22]. 

On 24 April 2017, WHO announced plans to make the RTS, S vaccine available in Ghana, Kenya, and Malawi through the countries’ national immunization programs [13]. Two years later, the pilot studies were underway. The vaccine manufacturer, GlaxoSmithKline, is providing up to 10 million doses of the vaccine, the countries’ ministries of health will lead the vaccine introduction through their national immunization programs, and WHO will provide scientific and technical leadership [23]. The vaccine formulation contains the most important surface resistance, the circumsporozoite protein (CS), which consists of an N-terminal region (RI) linked to the heparin sulfate proteoglycan, an intermediate region containing a tetraamino acid repeat (NPNA), and a GPI-anchored C-terminal region containing a thrombospondin-like domain (RII). The CS protein region included in the RTS, S vaccine contains the last 16 NPNA repeats and the entire flank C terminal. The hepatitis B virus surface antigen particle system (HBsAG) is used as a matrix carrier for RTS, S, which is 25% fused to the CSP segment [24]. The most recent study involving the RTS, S vaccine aimed at investigating the role of antibody isotypes other than IgG, which may also contribute to vaccine efficacy. Induction of peripheral blood IgA responses against vaccine antigens was observed, thus demonstrating the contribution of this immunoglobulin response to vaccine efficacy [25]. Furthermore, vaccination and naturally acquired immunity against microbial pathogens can have complex interactions that influence disease outcomes. Carlota Dobaño and collaborators observed that RTS, S immunization affects the acquisition of antibodies against Plasmodium falciparum antigens not included in the vaccine and that such responses have an impact on general protective immunity against malaria [26].

The second most advanced stage vaccine is the inactivated sporozoite vaccine from *P. falciparum*, PfSPZ [27]. In 2002, Sanaria Inc. was created to develop and market a sporozoite-based vaccine. The organization first developed the PfSPZ vaccine in 2003 [28], consisting of an intravenous vaccine with the radiation-attenuated *Plasmodium falciparum* parasite. The *P. falciparum* sporozoites were obtained manually by dissecting the mosquitos’ salivary glands [29], representing a challenge when it comes to the vaccine’s large-scale production. They were then irradiated and preserved to develop metabolically active and non-replicative (attenuated), aseptic, and purified sporozoites [29]. The first clinical trial of the PfSPZ vaccine involving a sample of eighty adults in 2011 demonstrated that the vaccine administered by subcutaneous or intradermal means was safe and well tolerated, in addition to triggering a low immunogenicity and efficacy performance [30].

There is general consensus that generating enough antibodies to block infection is a key challenge for induction protection in malaria vaccines [31]. Another recently discovered hurdle is epitope masking as an obstacle to antibody boosting after repeated administration of the attenuated *P. falciparum* sporozoite vaccine [32]. The authors showed that antibody titers to a key target, the repeat region of the *P. falciparum* circumsporozoite protein (PfCSP), plateaued after two immunizations in a clinical trial of the PfSPZ vaccine. It was determined that recall responses were inhibited by antibody feedback, potentially through epitope masking of the immunodominant PfCSP repeat region [32,33]. McNamara et al. reported that delaying a third dose until the vaccine antibody levels have dropped can alleviate cell suppression. Therefore, studies to evaluate the immunogenicity of the RTS, S vaccine were conducted with a fractional and delayed third dose of the formulation. The results suggested that the use of a fractional third dose of RTS, S (one-fifth dose volume), and changing the administration schedule from 0, 1, and 2 months to 0, 1, and 7 months resulted in very high levels of protection in adults [34,35]. These findings raised the hope of identifying the epitopes and corresponding antibodies involved in masking, B cell suppression, and impaired vaccine responses. 

Finally, there is a pre-erythrocytic malaria vaccine candidate R21/Matrix-M that was developed at the University of Oxford (Oxford, UK) and is currently manufactured by the Serum Institute of India (Pune, India). The ongoing safety, immunogenicity, and efficacy of R21/Matrix-M, and the number of malaria cases averted by this vaccine over 2 years of follow-up, following administration of the first booster dose have been reported [36].

Despite the advances, there is still a strong desire to find a highly effective vaccine against malaria. The question remains as to how effective and safe malaria vaccines can work to benefit those communities ravaged by malaria. Furthermore, there are challenges yet to be overcome regarding the development of such a vaccine, such as the extremely complex genome of *P. falciparum* and the limitations imposed by animal models. Scientists look forward to the discovery of new antigens and the improvement of platforms to expand the existing portfolio of vaccine candidates.

### 3.2. Pre-Erythrocytic Stage

Sporozoites travel through the blood in the pre-erythrocytic stage and infect hepatocytes which then undergo schizogony, the multiplication stage that precedes the invasion of red blood cells [37]. The main objective for developing a vaccine against the pre-erythrocytic stage is to inhibit hepatocyte infections and the development of the hepatic parasite, thus limiting the invasion of red blood cells [38]. Importantly, protection of sporozoites from malaria may depend on a fine balance of both innate and adaptive immune responses present in the pre-erythrocytic stages [39]. It is widely recognized that an effective, long-lasting malaria vaccine will need to induce robust antibody and T cell responses. Antibody responses to pre-erythrocytic stage antigens have been observed [40] and protected individuals may have titers of higher antibodies [41]. Regarding the adaptive immune response, it has already been reported that CD4^+^ T cells play a critical role in the response to the pre-erythrocytic stage of malaria [42,43]. Furthermore, the CD8^+^ T cells are recognized as the primary effector cells against the pre-erythrocytic stages, as observed in non-human primates [44]. 

Gardner et al. sequenced the *P. falciparum* genome [10]. The availability of the complete sequences of the *Plasmodium* genome allowed the development of live parasites attenuated by more precise and better-defined genetic manipulations. It was the strategy used in the patent US5112749A (Table 1), referring to a vaccine in which the immunogen is an attenuated entero-invasive bacterium that expresses a parasite epitope to elicit a protective immune response (humoral and/or cell-mediated) against *Plasmodium* infections. The epitope to be expressed is one of the CS proteins of the *Plasmodium* genus. The ability of the recombinant bacteria to trigger the production of antibodies against CS proteins was demonstrated in the invention, thus confirming the antigenicity of recombinant strains. The challenge was performed with *P. berghei* sporozoites by injection in the tail vein of mice and 80% of the immunized animals were protected. US 20160038580 A1 (Table 1) also provides a new nucleotide sequence and other constructs used for the expression of recombinant *P. falciparum* CS proteins in bacterial cells, such as *Escherichia coli*. The approach is also used in the AU2004309380B2 invention (Table 1), which relates to live genetically modified *Plasmodium* organisms and their use as immuno-effectors for vaccination purposes. The upregulated genes in infective sporozoites 3 and 4 (UIS3 and UIS4) are considered as essential for the early development of the liver stage [45]. This technology provides the first living, genetically modified *Plasmodium* organisms, the sporozoites UIS3 (-) and UIS4 (-), which infect hepatocytes, but are no longer able to establish infections in the blood stage and, therefore, do not lead to disease.

Despite these promising results, there are some disadvantages in using attenuated microorganisms as represented by need for the attenuation of *P. falciparum*. It has been shown that radiation required to generate an effective attenuated sporozoite must be precisely adjusted to meet minimum requirements [46]. Moreover, the attenuation process must also be precisely adjusted since the sporozoites exposed to high radiation levels do not induce protection, while parasites exposed to low levels induce infections [46]. Likewise, infections with different genetically attenuated *Plasmodium* sporozoites have been reported [47]. Since a single sporozoite in full development in the liver can give rise to blood infection and malaria symptoms, a vaccination based on the attenuation of *P. falciparum* sporozoites presents safety concerns that cannot be ignored [47].

An alternative strategy for the development of a malaria vaccine, based on the use of rodent *Plasmodium* parasites as a non-pathogenic vector for human immunization, was presented by the AU2013250814B2 invention (Table 1). It has been shown that *P. berghei* is capable of infecting human hepatocytes, which is necessary for the ideal presentation of the antigen, while at the same time being unable to cause an infection in the blood stage, thus ensuring the vaccine’s safety. The *P. berghei* mutant that expresses CS by *P. falciparum* (PbCSpf) was used and shown to maintain the primary characteristics of its wild-type counterpart while triggering a specific protective immune response against the *P. falciparum* challenge. This strategy opens other avenues for the design and production of additional vaccine candidates based on the same principle. While the development of a live vaccine could raise concerns regarding safety requirements, in addition to scale-up in the vaccine production, the EP1544211A1 invention (Table 1) describes a new *P. falciparum* liver sporozoite antigen referred to as Liver Stage Antigen-5 (LSA-5). This protein is highly antigenic and the prevalence of antibodies in individuals living in endemic areas is extremely high (roughly 90%). Immunization with LSA-5 induced protection against both challenges of *P. yoelii* (in mice) and *P. falciparum* (in *Aotus* monkeys). The results suggest that LSA-5 could be an important antigen candidate for an anti-malaria subunit pre-erythrocytic vaccine.

### 3.3. Blood Stage

The invasion of erythrocytes by *P. falciparum* involves a complex cascade of protein-protein interactions between the parasite’s ligands and the host’s receptors [48]. The Reticulocyte-binding proteins homologous of *P. falciparum* family (PfRh) are involved in binding and initiating invasive merozoite entry into erythrocytes [49]. In the invention WO2013108272A3 (Table 1), the authors described a receptor-blocking vaccine based on a combination of new erythrocyte-binding merozoite antigens that includes the PfRH (PfRH1, PfRH2a, PfRH2b, PfRH2b, PfRH4, and PfRH5). The vaccine targets erythrocyte-binding domains, blocking its interaction with its receptors and, therefore, inhibiting erythrocyte invasion. Another invention, US20190374629A (Table 1), provides a vaccine composition in which the PfRH5 antigen triggered antibody production resulting in at least 50% growth inhibitory activity (GIA) against a plurality of *Plasmodium* parasite blood-stage genetic strains. However, functional RH5 is only expressed in eukaryotic systems and new approaches are needed to stabilize the immunogen [50]. In particular, the invention provides rationally engineered modified PfRH5 antigens to produce improved stability and expression profiles while maintaining immunogenicity. The effectiveness of the modified PfRH5 antigens can be given in terms of their GIA, displaying up to 90% against blood-stage *Plasmodium* parasites. The inventors of US20140186402A1 (Table 1) provide an immunogenic composition for its use as a blood-stage malaria vaccine. The method consists of isolated or purified merozoites, or red blood cells infected with merozoites, treated with centanamycin or tafuramycin A in a mice model. A single dose of the composition is enough to protect against *Plasmodium chabaudi* and *Plasmodium vinckei*, without the need for an adjuvant. 

In the US20080026010 invention (Table 1), the authors describe the administration of a malaria parasite (*P. vivax*, *P. malariae*, *P. ovale*, and *P. falciparum*) with a modified gene to prevent infection in the host’s red blood cells. The *P. falciparum* depends on the acquisition of purines from the host for its survival in human erythrocytes [51]. Purine recovery by the parasite requires specialized transporters in the parasite’s plasma membrane (PPM) [51]. The invention deals with transgenic parasites without the PPM transporter. These attenuated strains can be grown and propagated in vitro under controlled conditions that require higher physiological concentrations of nutrients than those essential for the parasite. 

Alternatively, the use of a synthetic malaria vaccine instead of live parasites is described in the patent US4957738 (Table 1). This invention is a synthetic hybrid protein copolymer, used as a vaccine in humans against the *P. falciparum* asexual stages. The mixture of peptide compounds was injected into *Aotus trivirgatus* monkeys, inducing high antibody titers against the peptides and reacting with the *P. falciparum* parasite. The Colombian *A. trivirgatus* monkeys were immunized and challenged intravenously with blood cells infected with 5 × 10^6^ *P. falciparum* parasites obtained from an infected *A. trivirgatus* donor monkey. No parasites were detected in blood smear samples up to 90 days after challenge. The vaccine was also tested on human volunteers who were vaccinated two or three times with the synthetic protein copolymer. The volunteers were exposed to an experimental intravenous inoculation of red blood cells infected with one million fresh live ringed *P. falciparum* particles, resistant to grade chloroquine and with complete sensitivity to sulfadoxine and pyrimethamine, as are most wild Colombian strains. The composition was shown to induce complete and self-limited protection; three of the five vaccinated volunteers had mild infections with continual decrease in parasite count and full recovery on day 21. This synthetic hybrid protein, referred to as SPf 66, provides the first safe synthetic vaccine against the asexual stages. Moreover, a recombinant protein SE36, with 47 kDa, expressed in *E. coli*, was described in the invention US20040137512 as a highly effective formulation to prevent the *P. falciparum* growth in the blood considering human IgG3 antibodies are able to specifically bind to SE36 protein, thus blocking parasite growth.

### 3.4. Sexual Stage

Another type of approach targets vector control and parasite transmission strategies through the development of transmission blocking vaccines (TBVs) [52]. In this approach, the parasite’s transmission is interrupted by the host’s immune response to the parasite’s targeted proteins, such as pre-fertilization and post-fertilization antigens [53,54]. This type of vaccine aims to produce antibodies against the parasite and/or vector that will then interfere with the survival or virulence of the pathogen [55]. Thus, after the vector feeds on the infected and vaccinated host, the transmission of the pathogen is expected to be blocked [56]. The objective in malaria TBS is to prevent an individual from becoming infected with *Plasmodium* parasites by the *Anopheles* vector. As a result, the spread of malaria is expected to decline with reduction in the disease. The specific antibodies generated in the human host are passively ingested together with parasites when mosquitoes take a blood meal and will bind to the parasites, thereby preventing the progression of their sporogonic development [56]. Once inside the mosquito midgut, gametocytes rapidly emerge from the intracellular red blood cell environment to prepare for fertilization and are directly exposed to hostile immune components of the ingested blood [57]. Biologically, the sporogonic cycle is the most vulnerable part of the lifecycle because parasite numbers are very low, which makes this an attractive target for interventions [58]. 

The specific antigenic target, the surface antigen of *P. falciparum* 48/45 (Pfs48/45), was described as expressed by gametocytes [59] on the surface of the parasites’ sporogonic (macrogametes) stages. Pfs48/45 plays a key role in male gamete fertility and zygote formation, e.g., parasite fertilization [60], and the antibodies target conformational epitopes of Pfs48/45 that prevent fertilization [61]. This approach is present in the invention DK2763694T3, which describes a method of producing a cysteine-rich protein (CYRP) vaccine produced in bacteria derived from Pfs48/45 from *P. falciparum*. The WO2010036293A1 patent (Table 1) also describes the efficient and successful expression of the pre-fertilization antigen Pfs48/45 in high yields and appropriate conformation. A similar approach is described in the CN104736710A and US20150191518A1 inventions. In the CN104736710A patent, the authors used the *P. falciparum* P47 (Pfs47) or *P. vivax* P47 (Pfs47) surface antigens. The inventors proposed these proteins for blocking or reducing the infection by *P. falciparum* or *P. vivax* in *A. gambiae* or other anopheline mosquitoes and, thus, preventing the parasite transmission. In US20150191518A1 (Table 1), the authors reported a formulation capable of inhibiting the *P. falciparum* development inside the mosquito. This formulation includes a gamete surface protein, the *P. falciparum* gliding-associated protein 50 (PfGAP50). The inventors discovered that the emerging gametes of *P. falciparum* bind the complement regulator factor H (FH) following transmission to the mosquito to protect against the complement-mediated lysis by the blood meal [62]. PfGAP50 could be a candidate for TBVs since antibodies against PfGAP50 inhibit FH-mediated complement evasion of *P. falciparum*, resulting in the destruction of the malaria parasite by the human complement of the blood meal. *Anopheles stephensi* were artificially fed with neutral mouse antiserum and mouse Anti-PfGAP50 and the presence of the anti-PfGAP50 antibody reduced transmission rates by 68%.

Malaria transmission-blocking vaccines are advancing in clinical trials [63,64] and strategies for their introduction must be prioritized. Malaria TBVs are sometimes referred to as “altruistic” vaccines because they require herd immunity to reduce the incidence of malaria infection rates in the community, so this approach involves educational logistics and ethical challenges [65]. The benefits and implementation strategies of TBVs will need to be understood in advance, given that policies and actions must be coordinated among the stakeholders in many levels [66]. Therefore, if the TBVs are to succeed, the public will need to be aware of the importance of being immunized, not to mention that a large investment will be needed in the immunization policy.

### 3.5. Multicomponent and/or Various Stage Vaccines

Notably, one of the major challenges to developing a malaria vaccine is the parasite’s complex life cycle and its various stages of development. The first barrier that the malaria vaccine needs to control is the pre-erythrocytic phase, which requires protection against the infectious (sporozoite) form injected by mosquito and inhibits the development of parasites in the liver [67]. However, if some parasites escape the first barrier, a second one needs to act against the parasite blood stage (merozoite) to avoid multiplying within the erythrocytes [68]. Additionally, a third barrier needs to prevent the sexual phase and interrupt the transmission cycle by inhibiting the development of the parasites, since they are ingested by the mosquito along with the antibodies produced in response to the vaccine [55]. The third barrier (sexual parasite stages: gametocytes) concerns the transmission-blocking vaccines that may be involved as part of the multi-component or multi-stage vaccine strategy. This approach aims to eliminate the parasite and, at the same time, prevent the parasite’s resistance to anti-pre-erythrocytic or erythrocytic treatment, which is the focus of the inventions described below.

The idea to provide an additional immune response to the first, second, and third barriers against the *Plasmodium* infection was described in invention EP2923709A1 (Table 1). This technology involves new malaria vaccines composed of different recombinant proteins, in particular recombinant fusion proteins comprising several different antigens of the *P. falciparum* from the pre-erythrocytic, blood, and sexual stages. The pre-erythrocytic antigens consist of the PfCelTOS, PfCSP, and PfTRAP antigens; the blood stage antigens comprise at least one or more variants of Apical membrane antigen 1 (PfAMA1) or fragments thereof; and the iii sexual stage antigens include the ookinete antigen Pfs25 and/or the gamete/gametocyte surface protein Pf230C0 or variants or fragments thereof. The combination of recombinant proteins and fusion proteins outlined in this patent trigger a protective immunity that blocks infection, in addition to preventing the spread of the disease and interrupting the transmission of parasites. Rabbits were immunized and antibodies to rabbit antisera were purified by protein A chromatography. Sporozoite binding/invasion inhibition assays were performed to assess the ability of antisera directed against *P. falciparum* antigens to block the attachment and invasion of *P. falciparum* NF54 sporozoites to liver cells. The result was a 30% inhibition. Furthermore, there was an 80% inhibition in the GIA assay. Finally, membrane feeding assays were performed to assess the ability of antisera directed against *P. falciparum* antigens to block the transmission of *P. falciparum* NF54 from human to mosquito, and the transmission-blocking rates were between 80% and 100%. Using a similar approach, the authors of the invention CA2910322A1 (Table 1) proposed new recombinant fusion proteins against *P. falciparum* containing two or more different surface proteins introduced in at least two stages of the parasite’s life cycle. Immunofluorescence tests have confirmed that the induced antibodies bind specifically to native *Plasmodium* antigens. Further, functional tests showed specific parasite inhibition at each stage of the *Plasmodium* life cycle in a 30–100% range. Similarly, the EP2992895 invention relates to mixtures of recombinant proteins suitable as a human vaccine against the parasite *P. falciparum* comprising antigens derived from *P. falciparum* surface proteins of the pre-erythrocytic, blood and sexual stages of the parasite’s life cycle. This formulation contains TSR domain of the pre-erythrocytic antigen of *P. falciparum* CS protein, blood phase antigen of the apical membrane antigen (Pf AMA1), merozoite surface protein Pf Msp1-19, and peptides derived from Pf Rh5 and Pocs 25 antigen (EP2992895). Furthermore, the WO2017142843 invention (Table 1) provides polypeptides useful as antigens that are expressed in both the pre- and erythrocytic stages. The antigens can be used to induce cellular and humoral immune responses by administering the antigens in vaccine formulations or expressing the antigens using nucleic acid expression systems administered as a vaccine formulation. Notably, the polypeptides useful as antigens are the first pre-erythrocytic antigens of *Plasmodium* that induce sterile protection (100%) in mice against an infectious sporozoite challenge from *P. yoelii*.

## 4. New Approaches

The new pandemic of coronavirus disease (COVID-19) caused by the severe acute respiratory syndrome coronavirus 2 (SARS-CoV-2) presents new challenges to public health programs worldwide [69]. In countries heavily affected by malaria, COVID-19 could result in many years of hard-won gains being reversed [9]. The use of nanotechnology is a global trend for emerging vaccines. Nanoparticle glycoengineering has emerged as a powerful vaccine design tool [70] Synthetic glycosylation is fully characterized and stable and does not induce an unnecessary immune response to an antibody-based targeting moiety. Wilson et al. described a new synthetic glycoadjuvant, Man-TLR7, which is a random copolymer composed of monomers that either target dendritic cells (DCs) through mannose-binding receptors or activate DCs by means of Toll-like receptor 7 (TLR7). When conjugated with antigens, it elicits robust humoral and cellular immunity [71]. There was a significant increase in *Plasmodium falciparum* T-cell specific circumsporozoite protein (CSP) responses, expansion in the amplitude of the αCSP IgG response, and increased inhibition of sporozoite invasion in hepatocytes with CSP-p (Man-TLR7), as compared with CSP, formulated with MPLA/QS-21 -loaded liposomes, the adjuvant used in the most clinically advanced malaria vaccine [71]. 

Another recent advance has renewed prospects for the accelerated discovery of key malaria antigens, optimization of protein synthesis platforms through high-throughput immune screening approaches, reverse vaccinology, structural immunogen design, lymphocyte repertoire sequencing, and the use of artificial intelligence [72]. The wheat germ cell-free system (WGCFS) offers a eukaryotic alternative for expressing plasmodial proteins with wide application in malaria studies [73]. It was reported that malaria proteins produced by WGCFS have higher immunoreactivity to human immune sera as opposed to identical proteins produced in *E. coli* cell-free systems [74]. The disadvantage is that the WGCFS system lacks post-translational glycosylation mechanisms to produce glycoproteins, which play an important role in the vaccine-related immune response. Alternatively, glycoengineering could be used in cell-free glycoprotein synthesis (CFGpS), which perfectly integrates protein biosynthesis with asparagine-linked protein glycosylation [75]. Post-translational modifications are very important to consider when designing vaccines as they can be incorporated into T-cell epitopes, contrary to a classical dogma, in addition to enhancing humoral responses [76]. The presence or absence of small glycans on *Plasmodium* surface proteins, such as CSP, has the potential to influence the humoral response to a vaccine, such as RTS, S. For example, glycosylation may increase glycoprotein endocytosis by antigen-presenting cells and protect against excessive proteolysis of glycopeptides within the endolysosome by steric occlusion [77].

Immuno-profiling and reverse vaccinology approaches are essential to accelerating research and the development of new vaccine targets. A key message learned from these pioneering approaches is that it is crucial to select an expression system with the ability to produce many correctly folded recombinant malaria proteins without artificial glycosylation [73]. The development of a highly effective vaccine against malaria faces many challenges, including those associated with the identification of vaccine targets. The free expression of wheat germ cell-free systems can assist in testing hypotheses leading up to the development of a truly efficacious malaria vaccine.

## 5. Conclusions

As this review has made clear, the complexity of the malaria parasite cycle makes the development of a malaria vaccine a very difficult task. The parasite manages to circumvent the immune defenses by continuing the cycle. An approach to limit the immune system’s evasion mechanism by the *Plasmodium* is required to obtain a highly effective malaria vaccine. In this sense, the multi-component vaccine strategy based on blocking the *Plasmodium* infection shows great potential since it considers acting at three levels: (i) the pre-erythrocytic stage, (ii) the blood stage, and (iii) the sexual stage. As expected, the integrative approach using distinct *Plasmodium* antigens to limit each parasite’s life cycle could increase the malaria vaccine’s protection rate. The use of new technologies, such as nanoparticle glycoengineering, could play an important role in the development of emerging vaccines, such as a malaria vaccine. Concerns over the restricted experimental models used to analyze the anti-malaria vaccine candidates limit the global effort to develop an effective formulation that can be produced on a large scale. Considerable progress has been made in recent years in developing an effective vaccine against malaria. However, with the emergence of COVID-19 and efforts aimed at containing the pandemic, this cannot be allowed to restrict further advances toward making an effective vaccine commercially available.

## Figures and Tables

**Table 1:** Patents found in the patent databases: European Patent Office (Espacenet), United States Patent and Trademark Office (USPTO), United States Latin America (LATIPAT), and Patentscope -Search International and National Patent Collections (WIPO).

Registration Number	Invention Name	Inventors	Target Stage	Target Antigen	Database	Patent Date
*US5112749A*	Vaccines for the malaria circumsporozoite protein	Brey, Robert et al.	Pre-erythrocytic stage	Circumsporozoite recombinant proteins	USPTO, Espacenet	12 May 1992
*US20160038580A1*	Soluble recombinant *P. falciparum* circumsporozoite protein, use in vaccines, production methods and uses	Dutta, Sheetij	Pre-erythrocytic stage	Circumsporozoite recombinant proteins	USPTO	11 February 2016
*AU2004309380B2*	Live genetically attenuated malaria vaccine	Stefan, Kappe, et al.	Pre-erythrocytic stage	Live *Plasmodium* organisms genetically engineered	Espacenet	29 April 2010
*AU2013250814B2*	Rodent *Plasmodium* parasites as platforms for a whole-organism malaria vaccine	Mendes, Antonio Manuel Barbeiro et al.	Pre-erythrocytic stage	Live Plasmodium organisms genetically engineered	Espacenet	2 March 2017
*EP1544211A1*	LSA-5 pre-erythrocytic stage antigen of *P. falciparum*, an immunogenic composition comprising said antigen, and vaccines against malaria	Brahimi-Zeghidour, Karima and Druilhe, Pierre	Pre-erythrocytic stage	*P. falciparum* liver sporozoite antigen	Espacenet	22 Jun 2005
*WO2013108272A3*	Blood-stage malaria vaccine	Gaur, Deepak, et al.	Blood stage	Merozoite antigens	Patent scope, Espacenet	25 July 2013
*US20190374629A*	Thermostable variants of *P. Falciparum* PfRH5 that can be produced in bacterial cells	Draper, Simon, et al.	Blood stage	Modified homologous reticulocyte binding	USPTO, espacenet	12 December 2019
*US20140186402A1*	Blood Stage Malaria Vaccine	Good, Michael Spithill, Terry Lee, Moses	Blood stage	Isolated or purified merozoites	USPTO, espacenet	3 July 2014
*US20080026010*	Use of conditional *Plasmodium* strains lacking nutrient transporters in malaria vaccination	Ben Mamoun Choukri, El Bissati Kamal	Blood stage	Attenuated malarial parasite	Patent scope, Espacenet, USPTO	31 January 2008
*US4957738*	Protein copolymer malaria vaccine	Patarroyo, Manuel	Blood stage	Circumsporozoite proteins synthetic	USPTO, espacenet	18 September 1990
*US20040137512*	Malaria *Plasmodium* antigen polypeptide SE36, method of purifying the same and vaccine and diagnosis with the use of the resulting antigen	Horii, Toshihiro	Blood stage	Serine-repeat antigen of *P. falciparum*	Patent scope, Espacenet, USPTO, Latipat	15 July 2004
*DK2763694T3*	Preparation of a cysteine-rich protein	Andersen, Michael TheisenGorm	Sexual stage	Pfs48/45 recombinant protein	espacenet	16 April 2018
*WO2010036293A1*	Malaria vaccine	Kumar, Nirbhay Angov, Evelina	Sexual stage	Pfs48/45 recombinant protein	Patent scope, Espacenet,	3 June 2010
*CN104736710A*	Use of p47 from *P. falciparum* (pfs47) or *P. vivax* (pvs47) as a vaccine or drug screening target for the inhibition of human malaria transmission	Barillas-Mury, Carolina Veronica et al.	Sexual stage	*P. falciparum* P47 (Pfs47) or *P. vivax* P47 (Pfs47) surface antigens	espacenet	24 June 2015
*US20150191518A1*	Novel malaria transmission-blocking vaccines	Pradel, Gabriele et al.	Sexual stage	*P. falciparum* gliding-associated protein 50	USPTO, espacenet	9 July 2015
*EP2923709A1*	Multi-component-multistage malaria vaccine	Boes, Alexander et al.	Various Stage Vaccines	Recombinant fusion proteins	Espacenet	20 September 2015
*CA2910322A1*	Novel vaccines against apicomplexan pathogens	Boes, Alexander et al.	Various Stage Vaccines	Recombinant fusion proteins	Espacenet	20 October 2014
*EP2992895*	Three-component-multistage malaria vaccine	Fischer, Rainer et al.	Various Stage Vaccines	Recombinant fusion proteins	Espacenet	9 March 2016
*WO2017142843*	Novel antigen for use in malaria vaccine	AGUIAR, Joao Carlos	Various Stage Vaccines	Recombinant fusion proteins	Patent scope	24 August 2017
